# Three-Dimensional Printing of Large Ceramic Products and Process Simulation

**DOI:** 10.3390/ma16103815

**Published:** 2023-05-18

**Authors:** Tao Lin, Zhihao Zhao, Tao Wang, Ye-Tang Pan

**Affiliations:** 1 National Engineering Research Center of Flame Retardant Materials, School of Materials Science & Engineering, Beijing Institute of Technology, Beijing 100081, China; bhlintao@mail.tsinghua.edu.cn; 2 School of Materials Science & Engineering, Tsinghua University, Beijing 100084, China; zhao-zh19@mails.tsinghua.edu.cn; 3 Beijing Institute of Fashion Technology, Fashion Accessory Art and Engineering College, Beijing 100029, China; wangtaoforw@sina.com

**Keywords:** 3D printing, ceramics, material extrusion, fluid simulation

## Abstract

Ceramic 3D printing is a promising technology that overcomes the limitations of traditional ceramic molding. It offers advantages such as refined models, reduced mold manufacturing costs, simplified processes, and automatic operation, which have attracted a growing number of researchers. However, current research tends to focus more on the molding process and print molding quality rather than exploring printing parameters in detail. In this study, we successfully prepared a large-size ceramic blank using screw extrusion stacking printing technology. Subsequent glazing and sintering processes were used to create complex ceramic handicrafts. Additionally, we used modeling and simulation technology to explore the fluid model printed by the printing nozzle at different flow rates. We adjusted two core parameters that affect the printing speed separately: three feed rates were set to be 0.001 m/s, 0.005 m/s, and 0.010 m/s, and three screw speeds were set to be 0.5 r/s, 1.5 r/s, and 2.5 r/s. Through a comparative analysis, we were able to simulate the printing exit speed, which ranged from 0.0751 m/s to 0.6828 m/s. It is evident that these two parameters have a significant impact on the printing exit speed. Our findings show that the extrusion velocity of clay is approximately 700 times faster than the inlet velocity at an inlet velocity of 0.001–0.010 m/s. Furthermore, the screw speed is influenced by the inlet velocity. Overall, our study sheds light on the importance of exploring printing parameters in ceramic 3D printing. By gaining a deeper understanding of the printing process, we can optimize printing parameters and further improve the quality of ceramic 3D printing.

## 1. Introduction

Three-dimensional printing is an advanced material processing technology that has a wide range of applications in the field of traditional ceramic materials. However, due to its complicated preparation process, high consumption of consumables, long production cycle, and high cost, it has been challenging to produce parametric structures with highly complex geometric appearances and mechanical properties. This has hindered the development of the formation and creation of ceramic materials [1]. Fortunately, in recent years, 3D printing technology has become more mature and has demonstrated the dual characteristics of high precision and high forming freedom. Its high printing precision overcomes the typical geometric limitations of traditional ceramic manufacturing technology and allows ceramic materials to be more closely combined with art and science [2].

For advancing the field of ceramic materials, 3D printing is a powerful tool. As the technology continues to evolve, we can expect to see more breakthroughs in the creation of complex ceramic structures, leading to new possibilities for artistic expression and innovative applications in various fields. Additive manufacturing technologies for producing three-dimensional ceramic parts include inkjet printing [3,4,5], selective laser sintering (SLS) [6,7], stereolithography (SLA) [8,9], laminated object manufacturing (LOM) [10,11], and direct writing extrusion-based technologies [4,12,13]. In the 3D printing process, the 3D ceramic molding process is transformed into a discrete accumulation process from point to line, and from line to surface [14]. This involves slicing the three-dimensional model into two-dimensional paths with specific shapes [15], and then stacking the ceramic mud layer by layer along the paths to achieve three-dimensional molding [16]. The clay printing unlike the materials used in conventional FDM printing technology [17], the unstable nature of clay and the imprecise pressure from the air pump can lead to the insufficient adhesion of printing materials and poor molding quality. This can result in a low printing success rate. Therefore, the clay printing and molding process requires parameter testing and numerical simulation of the printing equipment to ensure optimal performance. In conclusion, 3D printing offers a unique approach for creating complex ceramic structures, but it also presents some challenges that must be addressed. By continuously improving and optimizing the printing process, we can unlock the full potential of 3D printing for ceramic materials and expand its applications in various fields [18].

Revelo [19] utilized direct ink writing (DIW) technology to 3D print ceramics made from kaolin clay. The printed samples underwent compression, thermal stability, and density testing, as well as Weibull analysis, to assess their quality and performance. Moreover, the rheological behavior of the slurry during the printing process, along with the process parameters, was studied to comprehend key manufacturing factors and optimize production processes. Benedikt et al. [20] conducted a study on the influence of ceramic clay formula parameters (alumina) on the characteristics of ceramic paste and green bodies. They determined the optimal printing and sintering parameters based on their findings. Abhinav et al. [21] used a ceramic clay printer to investigate the feasible parameter area for ceramic 3D printing using extrusion. They studied the effects of extruder height, layer thickness, printing speed, and air pressure on the surface quality of the printed samples. Zhang Min’s group [22] conducted parameter simulation research on screw extrusion and direct writing extrusion. They used velocity distribution, shear rate, and pressure distribution simulations to conclude that direct writing 3D printing is suitable for printing high-viscosity materials, while screw extrusion is beneficial for printing multiple materials. He Mingteng et al. [23] simulated the extrusion molding process of clay using ANSYS CFX. They identified the main and secondary factors that affect the stress state in the extrusion blank, such as the length of the shaping section, inlet pressure, moisture content, and extrusion cone angle. They also obtained the optimal combination of these influencing factors. Salah-Eddine et al. [24] investigated the effects of printing conditions and annealing on the Z-directional porosity and tensile behavior of a 3D-printed polyetherimide material. Through their experiments, they demonstrated that printing speed is the most significant factor affecting the tensile properties and density of Z-directional, 3D-printed parts. Anouar El Magri [25] systematically studied nozzle temperature (T), printing speed (S), and layer thickness (L), and optimized the output responses of Young’s modulus, tensile strength, and crystallinity. As ceramic 3D printing technology continues to advance, we strive to achieve finely crafted ceramic devices with desired structures and qualities through the meticulous control of the ceramic forming process. However, accomplishing this goal requires a quantitative characterization of the printing parameters and quality testing [26]. Currently, research on the printing materials and models for ceramic 3D printing has matured, but there are still gaps in the study of these printing parameters. The print molding quality can be significantly impacted by various factors during the printing process, such as the type of 3D printer, printing speed, printing path, extrusion flow rate, nozzle shape, and size, among other factors [27]. Chen et al. [28] conducted experimental and numerical studies to investigate the impact of layer spacing and nozzle distance on the interlayer bonding strength of 3D-printed limestone and calcined clay-based materials. Their findings suggest that prolonging the time interval between two layers leads to a reduction in bonding strength, while increasing the nozzle distance has only a limited effect on it. Mehdi’s group [29] investigated the influence of three process parameters (distance between nodes on the printing path, nozzle-to-substrate distance, and delay time) on the interlayer bonding strength of clay-based materials produced via additive manufacturing. Their study revealed that the periodic manipulation of the nozzle distance functions similarly to compaction in traditional concrete pouring techniques, resulting in a 41% increase in the average interlayer bonding strength under shear stress, ultimately enhancing the quality of the printing. Compared with other printing materials, the flow state of ceramic clay is more uncertain, and the control of the finished product quality is still unstable. Therefore, systematic experimentation and simulation are needed to obtain the optimal mechanical parameters with regard to forming quality, where the inlet speed and mechanical screw speed of ceramic clay are the two core parameters. The inlet speed pertains to the velocity at which materials enter the printing area through the extruder’s feed port. If the inlet speed is too swift, it can result in defects on the printed object’s surface or to nozzle blockage. Conversely, if the inlet speed is too sluggish, it may lead to an extended printing time and higher expenses. Thus, controlling the inlet speed accurately is crucial in producing high-quality printed objects. Additionally, the mechanical screw speed configuration can impact the printing speed, the surface quality of the printed object, and other factors. The mechanical screw plays a vital role in 3D printers by feeding materials from the printer’s feeder to the nozzle. A mechanical screw speed that is too high may cause uneven material extrusion or nozzle clogging. On the other hand, a mechanical screw speed that is too slow can result in a slower printing speed and increased costs. Therefore, it is essential to set the inlet speed and mechanical screw speed appropriately during 3D printing and adjust them according to different materials and printer models to achieve optimal printing outcomes.

Overall, research on the parameters for ceramic 3D printing is an active area of investigation, as this research is critical to achieving high-quality printed products. By understanding the effects of various parameters on the printing process, we can optimize the process and develop new applications for ceramic 3D printing. The simulation of the internal flow and pressure field state of the nozzle can help us to understand the behavior of the clay material during the printing process, and the gradient control of the inlet velocity and screw speed can optimize the printing parameters and improve the molding quality. By analyzing the point velocity parameters of the printing outlet, the study can provide insights into how the printing speed affects the adhesion and accuracy of the printed layers. Overall, this study can contribute to the advancement of 3D printing technology for ceramic materials and expand the possibilities of artistic and functional applications of ceramic products.

## 2. Experimental Section

### 2.1. Experimental Principle

**Figure 1:**
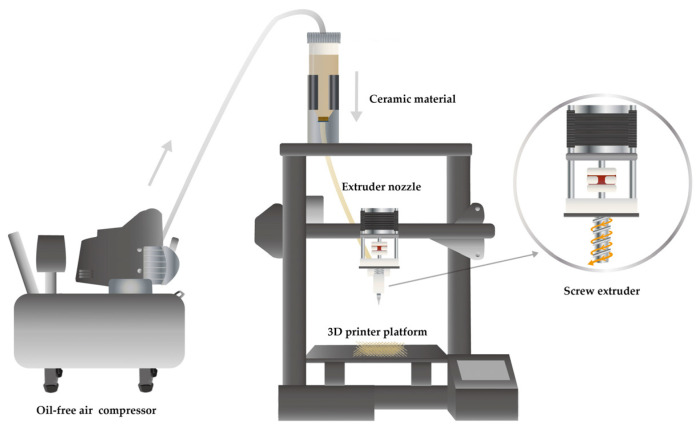
Schematic diagram of screw extrusion direct writing equipment.

The 3D printing equipment used in this study combines extrusion direct writing technology (DIW) [30] with FDM technology to create an air pump screw extrusion direct writing technology specifically designed for clay. The enhanced pottery clay printing and molding equipment, in combination with FDM technology [17], utilizes an air pump to compress the clay into a pipeline for storage before extruding it through a mechanical screw pressure bar. By designing the nozzle size to have varying diameters, the pottery clay can be extruded while maintaining a certain level of adhesiveness and toughness. The implementation process is depicted in Figure 1. The digital design printing program converts the three-dimensional model slice into a two-dimensional path, and the printing nozzle moves along this path to build the ceramic mud stack and create the desired ceramic mud blank. The specific implementation steps are as follows: (1) The clay 3D printer identifies the modeling slice file and creates a printing path program [15]. (2) Air pressure from the air pump pushes the clay into the pipeline, where it is stored. (3) The screw rotates to drive the extrusion of the clay. (4) The pipe diameter gradually decreases, and the clay is extruded in strips. (5) The program path is followed to stack and mold the clay.

### 2.2. Experimental Process

#### 2.2.1. Three-Dimensional Printing Experiments

**Figure 2:**
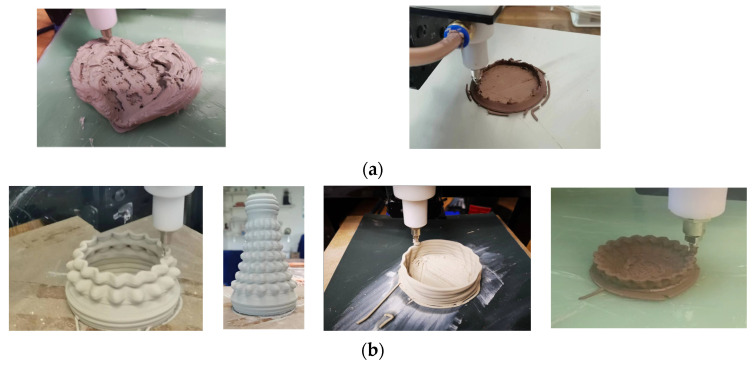
(**a**) Failed print sample; (**b**) Successful printed object.

The operation of the clay 3D printer can be divided into five steps: (1) The preparation of the clay raw materials involves mixing clay with water in a specific ratio to achieve the desired water content. (2) Establishing a printing model requires the use of SolidWorks (Dassault Systemes SolidWorks Corporation, Waltham, MA, USA) or similar software to design and control the size and details of the model [31]. The 3D model can be created from scratch or imported from an existing design file. The slicing software Cura is used to set the slicing and printing parameters [32]. In this study, the basic printing parameters included a layer thickness of 0.6 mm, a printing speed of 30 mm/s, a nozzle temperature of 0 °C (referring to the temperature of the applied heat source), and a nozzle diameter of 1.5 mm. (3) The slice file is imported into the printer to carry out the steps of leveling, connecting, mud discharging, speed regulation, and so on, until the mud discharging is stable. (4) A quality inspection is performed to ensure that the ceramic mud is evenly discharged and the finished product has clear lines and complete details. (5) Drying and glazing: once the printing is complete, the printed object needs to be dried before it can be fired in a kiln. The drying process can take several hours or even days, depending on the size and complexity of the object. After the object is dried, it can be sanded, smoothed, or glazed to achieve the desired finish. The finishing step is important for improving the aesthetic appeal and durability of the printed object. Figure 2a,b demonstrate how the extrusion speed is greatly affected by both the water content and air pump input pressure, resulting in noticeable differences in the molding quality of the printed clay.

#### 2.2.2. Sintering Process

**Figure 3:**
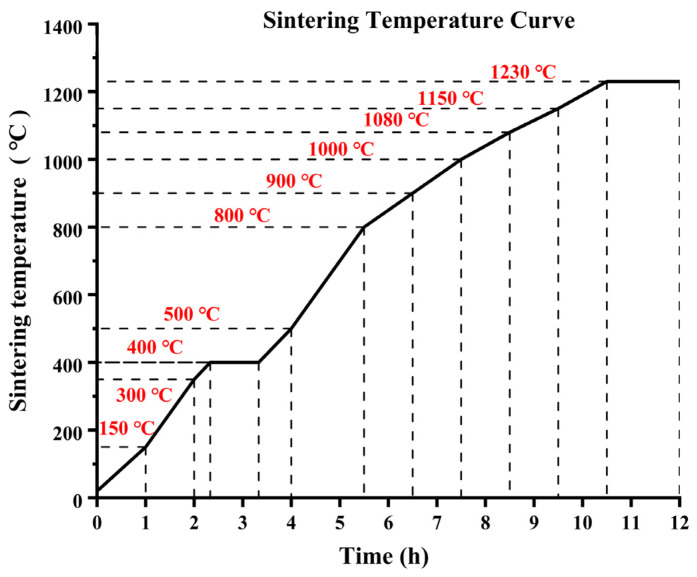
Ceramic sintering temperature curve.

The heating curve is shown in Figure 3. We developed a sintering method that corresponds to the TG-DTG curve. The temperature ranged from 25 to 150 °C in the drying stage, while it was 150–350 °C in the initial drainage and evaporation stage. Between 350 and 500 °C, we exclude large water molecules, with 400 °C being the point where the clay was most susceptible to cracking. The temperature range of 500–800 °C was where we removed the crystallization water molecules. At 800 °C, burning off water molecules in the clay’s molecular structure causes the clay body to shrink due to water removal. Between 800 and 900 °C, organic matter oxidizes and combusts, and the same occurs between 900 and 1000 °C. At 1000–1080 °C, sintering begins, while at 1080–1150 °C, the glaze starts to melt and crystals start to preliminarily react. Between 1150 and 1230 °C, we could see the reaction of glaze crystals and high-temperature synthesis. The sintered product was then cooled in the furnace.

#### 2.2.3. Material Characterization

**Figure 4:**
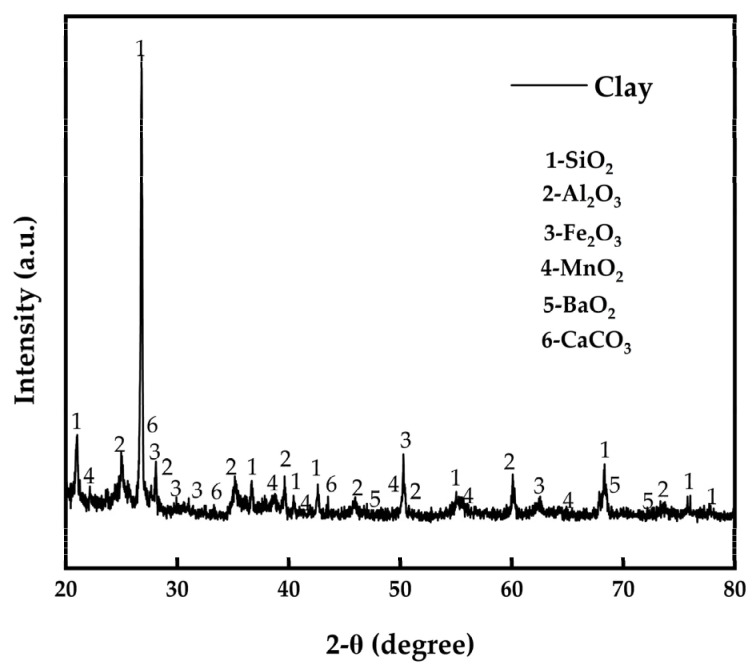
XRD analysis spectrum of clay composition.

The X-ray diffraction (XRD) design of the samples was measured using a Cu radiation diffractometer (40 kV and 15 mA) from 20° to 80° (2θ) at a scan rate of 2°/min with 0.02° steps. The component ratios of the raw materials for pottery clay were as follows: kaolin accounted for 41%; feldspar accounted for 35%; washed kaolin accounted for 10%; potassium feldspar accounted for 8%; and quartz accounted for 6%. XRD analysis was carried out on the mixed raw materials for pottery clay. Figure 4 shows that the main components present were SiO_2_, Al_2_O_3_, Fe_2_O_3_, MnO_2_, BaO_2_, and CaCO_3_.

**Figure 5:**
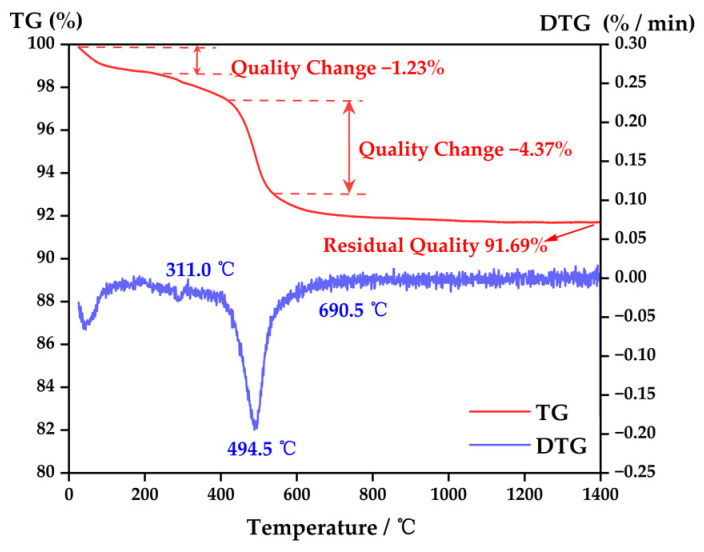
TG–DTG curve of clay.

The samples were measured using a 449F5 thermal analyzer instrument (Netzsch, Sta, Selb, Germany) for thermogravimetric analysis (TGA) in the temperature range from 25 to 1400 °C with a heating rate of 3 °C/min under an air atmosphere. The TG-DTG curve of the clay is presented in Figure 5. Based on the analysis of the curve, it can be observed that the clay exhibited an exothermic peak at approximately 400 °C, leading to a mass loss of 4.37% on the TG curve. As a result, insulation was required during the 400 °C stage. Once the temperature reached 690.5 °C, the mass of the ceramic body remained relatively stable, indicating that the moisture in the clay had completely evaporated before this temperature was reached. To prevent defects such as cracking and collapse during the sintering process of the ceramic body, it is crucial to meticulously adjust the heating rate and insulation time for each stage, ensuring that each stage lasts for around 1 h.

#### 2.2.4. Simulation Process

**Figure 6:**
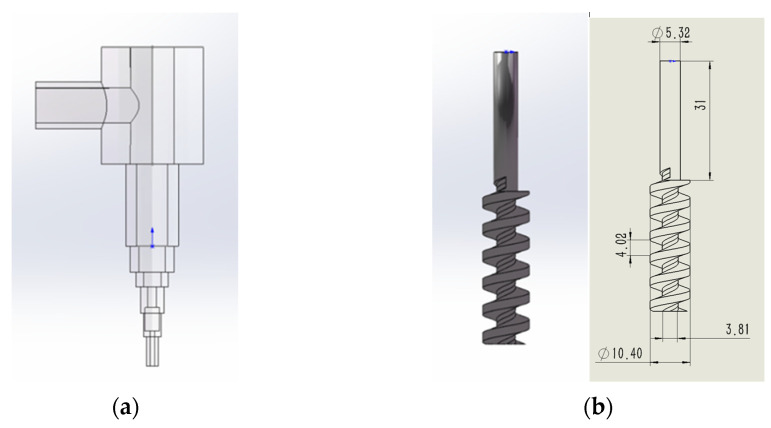

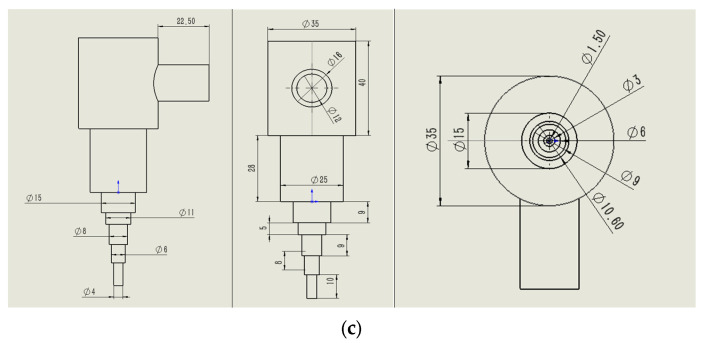
(**a**) Pipe model of extrusion nozzle. (**b**) Screw model of extrusion nozzle. (**c**) Model parameters of extrusion nozzle (three views).

Firstly, we observed the nozzle structure entity, disassembled the parts and tested the parameters, and used an electronic vernier caliper to measure the size and model. Using SolidWorks modeling software, two parts, the nozzle and the rotating screw, were established for coaxial assembly. The three-dimensional diagram and specific parameters of the model are shown in Figure 6a–c. A flow simulation plug-in was used for fluid simulation. We set the basic parameters of clay as follows: the density was 1285 kg/m^3^; the consistency coefficient was 1.2 Pa·s; the specific heat was 840 J/(kg × K); the thermal conductivity was 1 w/(m × k); the viscosity adopted the power law model; and the temperature was set to room temperature. The gravity condition was set to the Y direction (the direction of the flowing clay) with a gravity acceleration of 9.81 m/s^2^. The simulation range was set to a rectangular parallelepiped that fully encompassed the fluid domain. The boundary condition was set to a gradient that specified the inlet velocity and total pressure of the inlet cover surface as atmospheric pressure. The rotational condition was applied to the cylindrical part of the screw, which was defined as the rotational domain, with a gradient specifying the screw’s rotational speed. Finally, the output was set to include the average velocity and static pressure values of the fluid in the chamber. The experimental parameters of the simulation model were set according to a certain gradient, with the inlet speed and screw speed as shown in Table 1, creating nine groups of parameters that were designed by crossing 3 × 3. Then, we could conduct the simulation analysis.

## 3. Results and Discussion

Fused deposition modeling (FDM) is the most commonly used 3D printing method, based on an extrusion process that selectively distributes materials through nozzles or holes [33]. By exploring the material and printing process parameters of FDM technology, the printing results can be optimized [34]. FDM usually involves the use of 3D design software to create a digital design, which is then divided into a series of laminated data. These data are transmitted to the printer, which reproduces the design layer by layer until a complete model is obtained [35]. Manikandan et al. [36] discovered that the geometry of the nozzle has a significant influence on the 3D printing of clay-based materials. They observed that circular nozzles produce less surface roughness and contour deviation for cylindrical structures, whereas square nozzles result in higher compression strength, but also relatively higher contour deviation and surface roughness.

### 3.1. Three-Dimensional Printing

**Figure 7:**
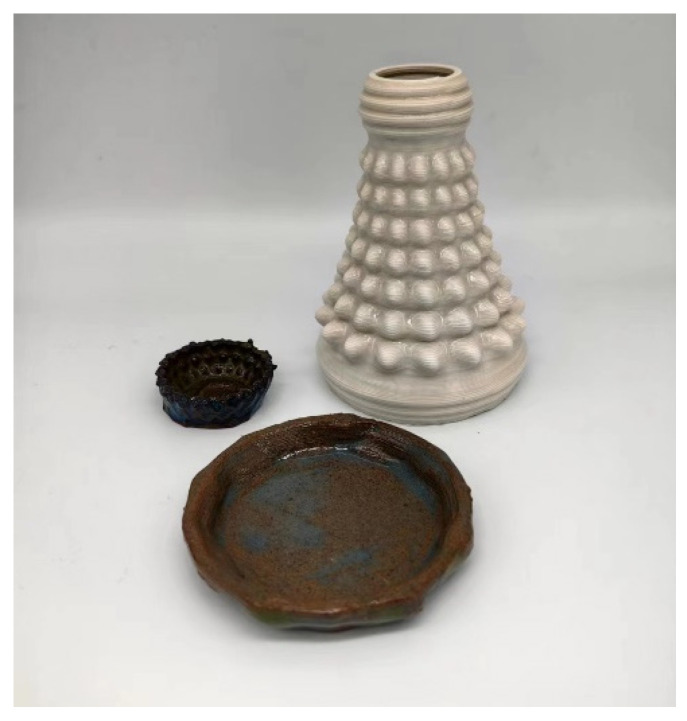
Sintered products.

Figure 7 showcases the sintered products, which completely preserved the overall shape and microscopic details of the clay blank, providing indirect evidence of the print molding quality of the forming process. Through the combination of the experimental processes, it was observed that the control of the moisture content in the clay was critical during the actual printing process. If the moisture content was too high, the clay would collapse, making it impossible to stack and form the desired shape. Conversely, if the moisture content was too low, the clay would dry up, resulting in the formation of lumps that are challenging to adhere and shape. After several adjustments, it was discovered that the optimum moisture content should be maintained between 25% and 27%. For instance, to mix 1 kg of clay, 60 g of water should be added, and the mixture should be stirred for 3–5 min at a speed of 100 r/s until it is uniformly distributed. The mixture can then be loaded into the printing material cylinder for experimentation. To ensure the stability of the clay extrusion during the printing process, the optimal input pressure for the air pump should be controlled at 0.2–0.4 MPa, ensuring that the extruder nozzle is not blocked and has sufficient printing material.

Additionally, the size of the nozzle should also be carefully chosen to ensure the desired level of detail and accuracy in the printed object. A smaller nozzle size can allow for more precise details, but can also increase the risk of clogging and may require slower printing speeds. It is also important to regularly clean the printer nozzle during the printing process to prevent blockages and ensure consistent extrusion. Finally, the drying and glazing processes after printing should be carefully controlled to avoid cracking and ensure a smooth and even finish. With careful attention to these factors, high-quality and complex ceramic crafts can be successfully 3D printed using clay.

### 3.2. Simulation

#### 3.2.1. Simulation Principle

The aim of this simulation was to study the influence of the design parameters on the flow characteristics of ceramic clay during the printing process. The simulation results show that the design of the nozzle size, shape, and angle can affect the flow state and pressure distribution of the ceramic clay. By adjusting the design parameters, the flow state and pressure distribution of the ceramic clay can be optimized, thereby improving the quality of the printed ceramic crafts. In addition, the simulation can also provide guidance for the actual production process, such as adjusting the printing speed, optimizing the nozzle design, and improving the quality of the printed ceramic products [37]. By combining the simulation results with experimental data, the printing process can be optimized to achieve the desired product quality and reduce the production cost. Yang’s group [38] conducted a simulation and analysis of the extrusion-based 3D printing process using the computational fluid dynamics (CFD) method. They developed a material extrusion process model that relied on a pressure-driven system and confirmed the accuracy of the simulation results. By comparing the average flow rate of the printing clay under different air pressures in experiments, they were able to precisely predict the flow rate. By adjusting the printing speed and layer height based on the simulation, they were able to control the shape and size of the extruded filament. Tu et al. [39] conducted a simulation of the velocity field of a slurry as it passed through a nozzle. In their simulation, they took into account various factors such as the material properties of the printed slurry, the dimensions of the extrusion tool, and process parameters. This simulation model has the potential to predict the velocity field and shape of extruded filaments in the air. Based on their study, we simulated the velocity field of the ceramic clay passing through the nozzle while taking into account various factors such as the material characteristics of the printing clay, the size of the extrusion tool, and process parameters. By doing so, we were able to accurately predict the velocity field and shape of the extruded material in the air.

#### 3.2.2. Simulation Result

**Figure 8:**
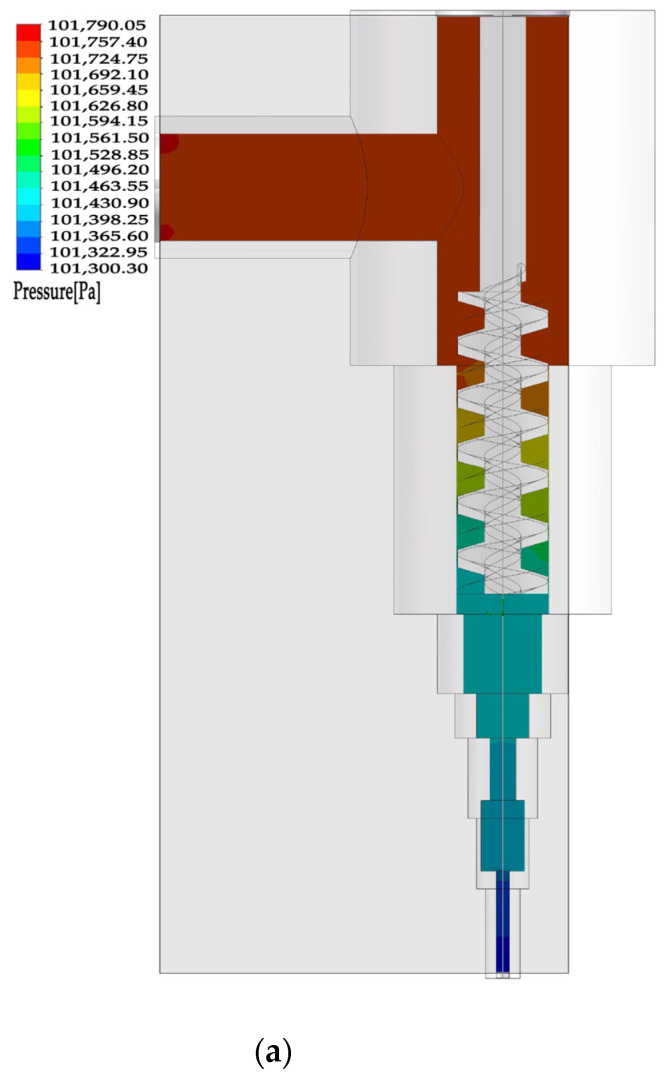

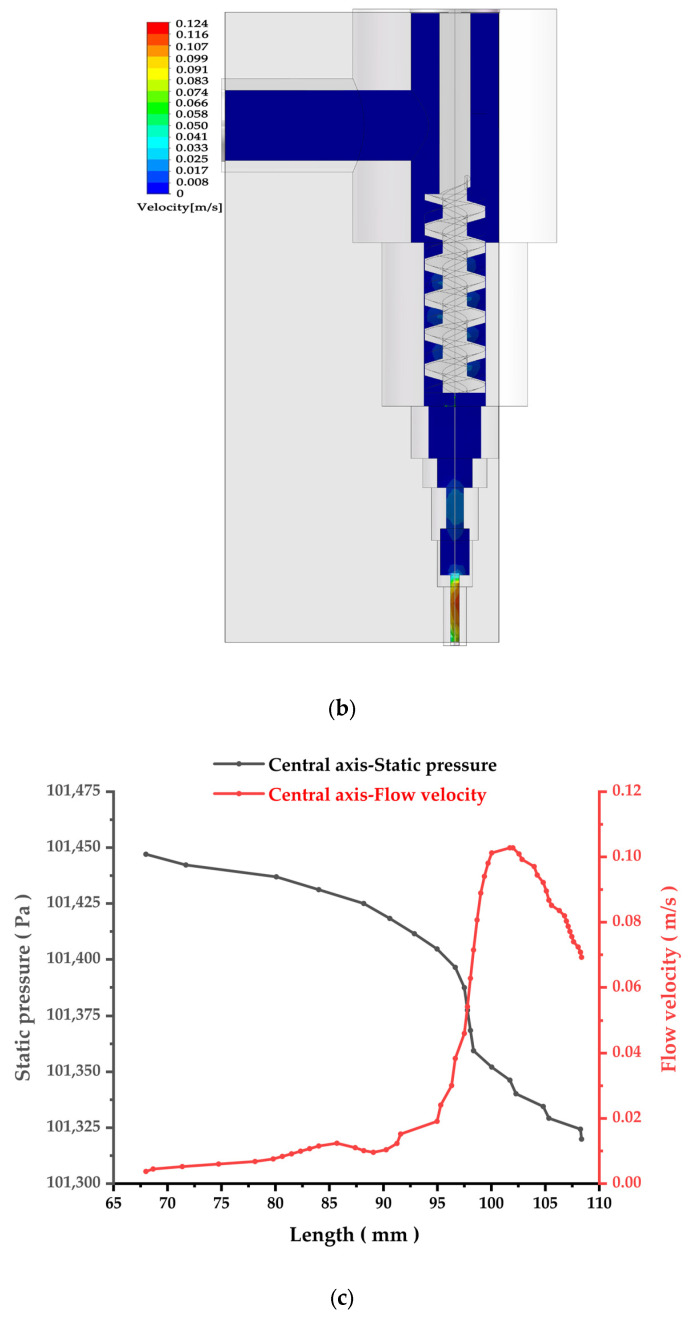

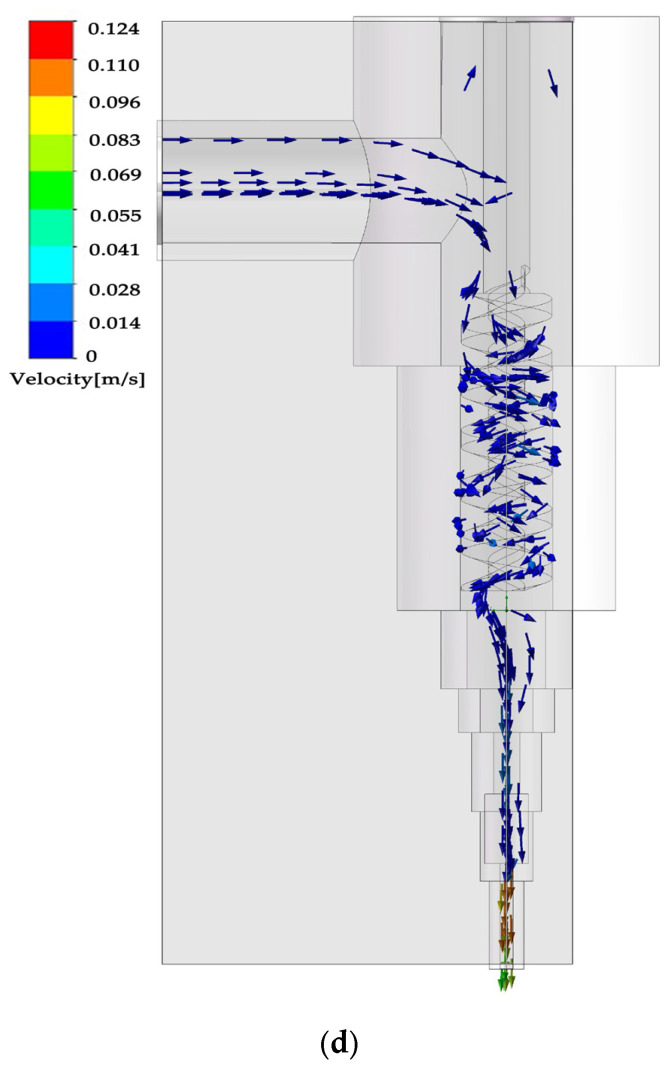
(**a**) Pressure field inside the model. (**b**) Internal velocity field of model. (**c**) Distribution of fluid pressure and velocity in the central axis. (**d**) Dynamic simulation flow tracing.

As shown in Figure 8a–c (there were nine groups, and the second group was selected for illustration), it can be seen that the pressure field in the upper clay storage place was not obviously changed due to the buffering effect of the pipeline. Under the mechanical rotation power of the mechanical screw, the pressure field changed, which drove the ceramic clay to move. With the extrusion of the screw and the narrowing of the pipeline diameter, the flow speed of the ceramic clay increased, forming a strip-shaped ceramic clay extrusion at a certain speed. The fluid simulation function of the flow simulation was used for the dynamic simulation, and the flow process tracing is shown in Figure 8d.

The simulation results show that the flow state of the clay in the nozzle was affected by the inlet velocity and the screw speed. When the inlet velocity was constant, as the screw speed increased, the flow rate of the clay in the nozzle also increased and the pressure inside the nozzle increased. When the screw speed was constant, as the inlet velocity increased, the flow rate of the clay in the nozzle also increased and the pressure inside the nozzle also increased. The simulation also shows that the diameter of the nozzle has a significant impact on the flow state of the clay. As the diameter of the nozzle decreased, the flow rate of the clay in the nozzle decreased and the pressure inside the nozzle increased. These results can provide a theoretical basis for optimizing the parameters of the clay 3D printer to improve the print molding quality of ceramic crafts.

The nine groups of results set according to the gradient were compared and analyzed, and the results are shown in Table 2 (the data unit in the table is m/s).

The results of the fluid simulation accurately represent the behavior of the entity being studied. A quantitative analysis of the outlet velocity revealed that the inlet velocity had a significant impact on the outlet velocity, and the two were positively correlated with a proportional coefficient of approximately 700. However, the rotating speed of the screw had no significant impact on the outlet velocity. Based on an analysis of the entity, it was found that the deviation of the results was mainly due to the limitations of the model and the approximations made in the rotating process. In reality, the clay model is introduced through an air pump and enters the nozzle via a slender pipe, which may result in differences between the actual velocity and the inlet velocity of the model. The collision of the fluid may also affect the actual flow. In the modeling of the rotating screw, some fluid may move down through the gap instead of being rotated and squeezed by the screw, which is different from the actual situation. This may result in the influence of screw rotation on the outlet velocity being obscured by the inlet velocity.

## 4. Conclusions

Based on a specific experimental process, we found that one of the most important parameters that affects print molding quality is the printing outlet velocity, which refers to the extrusion speed of the ceramic clay. If the speed is too fast, it is easy to produce printing defects and the ceramic clay is prone to block the outlet, which can cause subsequent printing to be impossible. If the printing speed is too slow, it will cause the ceramic clay to be unable to form a good bond with the bottom and produce printing breakpoints, resulting in the printer’s inability to continue stacking and forming. The two core mechanical parameters that affect the outlet velocity are the inlet velocity of the clay and the internal mechanical screw rotational speed, which jointly control the flow velocity and bonding state of the clay in the pipeline. Therefore, research on the parameters of clay 3D printing can start from these two mechanical parameters and explore their numerical relationship with the outlet velocity, thus controlling the quality of the printed product.

This study utilized 3D printing and molding equipment based on FDM technology to conduct modeling and fluid simulation analysis on the clay outlet parts. Based on the 1:1 modeling of the entity and making approximate adjustments to the structure of the rotating screw, nine sets of simulation models were obtained by adjusting the inlet velocity and screw speed. It was discovered that the extrusion velocity of clay was approximately 700 times the inlet velocity at an inlet velocity of 0.001–0.010 m/s. However, due to the limitations of the model, the influence of screw speed was obscured by the effect of inlet velocity. In future research, simulation details and the model can be refined. Additionally, experimental parameters can be combined with actual printing parameters and operational verification parameters can be selected to enable a combination of simulation and actual verification, providing a more robust theoretical basis for the integrated molding technology of 3D-printed clay blanks. This can ultimately lead to the realization of automatic ceramic molding with high precision and freedom.

## Figures and Tables

**Table 1:** Simulation group.

Group Setting	Screw Speed of 0.5 r/s	Screw Speed of 1.5 r/s	Screw Speed of 2.5 r/s
Inlet velocity of 0.001 m/s	1#	2#	3#
Inlet velocity of 0.005 m/s	4#	5#	6#
Inlet velocity of 0.010 m/s	7#	8#	9#

**Table 2:** Test results of outlet velocity.

Inlet Velocity (m/s)	Screw Speed of 0.5 r/s	Screw Speed of 1.5 r/s	Screw Speed of 2.5 r/s
Inlet velocity of 0.001 m/s	0.0754	0.0751	0.0753
Inlet velocity of 0.005 m/s	0.3448	0.3449	0.3448
Inlet velocity of 0.010 m/s	0.6828	0.6762	0.6813

## Data Availability

Not applicable.
